# Population-based cancer registries: a gateway to improved surveillance of non-communicable diseases

**DOI:** 10.3332/ecancer.2020.ed95

**Published:** 2020-01-16

**Authors:** Les Mery, Freddie Bray

**Affiliations:** Cancer Surveillance Section, International Agency for Research on Cancer, 150 cours Albert Thomas, 69372 Lyon Cedex 08, France

**Keywords:** cancer registries, surveillance, cancer, non-communicable diseases, Sustainable Development Goals

## Abstract

Timely and accurate data on health enable policymakers to make informed decisions that can reduce the burden and suffering from disease. Yet many LMICs are not able to adequately collect the health indicators necessary to track progress in the Sustainable Development Goals (SDG) at present, and a major investment in primary data collection is needed. We argue that cancer surveillance, with an established history of international standards and best practices, represents a feasible entry point in the development of surveillance programmes for NCDs. The International Agency for Research on Cancer (IARC) has served to support population-based cancer registries (PBCR) since its inception over 50 years ago. Based on this longstanding experience and collaboration with PBCR worldwide, IARC and other key partners implemented the Global Initiative for Cancer Registry Development (GICR, http://gicr.iarc.fr/) as a new way to deliver capacity-building in cancer surveillance. We describe some of the critical aspects of the GICR and the prospects of a step-change in the quality and use of cancer data over the next years. Ultimately, the decision on how to proceed resides with countries. The cancer and NCD burden will not be tackled without committed and sustainable action by governments.

Timely and accurate data are rightly valued in healthcare, providing policymakers with the means to make informed decisions that can save lives and reduce suffering. In order to prioritise the most effective interventions, evaluate their impact, and where necessary, refine policies, relevant, routinely-collected data must be at hand.

Perhaps such an opening statement is self-evident and would go unchallenged by decision makers and the public health community at large. Yet a situation analysis of the availability and quality of primary data from national surveillance systems reveals a staggering data inequity worldwide that hampers the ability of many governments to effectively plan healthcare and monitor national targets. The World Health Organization (WHO), in assisting Member States to meet the reporting requirements of the Sustainable Development Goals (SDGs), estimate that one-third of Member States have no primary data on over half of the 18 relevant indicators required for monitoring the

SDGs [1]. Cause-of-death data, as a core indicator available from national vital registration systems, is complete and of high quality in only one in four Member States [2]. The data deficits are predominantly in low- and middle-income countries (LMICs), particularly the WHO African and South-East Asia regions, where the completeness of mortality data is around 6% and 10%, respectively.

Recent papers attest to the overwhelming necessity to invest in primary data collection in LMICs to aid local decision-making [3], and to break the chain of dependence on global estimates that may arise from a systematic lack of investment in national information systems [4]. There clearly remains a need to better connect various efforts to improve data collection on the ground, and significantly invest in global coordination and implementation.

Is it appropriate to fast-track the local collection of cancer data? We argue below that cancer surveillance, with an established history of international standards and best practices, represents a feasible entry point in the development of surveillance programmes for NCDs. Certainly the current and future impact of cancer is unrefuted in public health and economic terms; we are in the midst of a disease transition that will see cancer become the leading cause of premature death in every country of the world in this century and local data for local action is imperative worldwide. The essential elements of cancer surveillance need to be first integrated into surveillance systems for NCDs; at the same time, different measures and strategies of cancer surveillance need to be linked to components of cancer control [5].

The specifics of cancer data collection via population-based cancer registries (PBCR) are unique [6]. The International Agency for Research on Cancer (IARC), the specialized cancer organization of the WHO, has served to support cancer registration since its inception over 50 years ago. Based on this longstanding experience and collaboration with PBCR worldwide, IARC and other key partners implemented the *Global Initiative for Cancer Registry Development* (GICR, http://gicr.iarc.fr/) in 2012, as a new way to deliver capacity-building in cancer surveillance. Based on the essential factors for cancer registries described below, countries are approached to partner in a data quality improvement plan.

The methods to collect cancer data via PBCR are well documented and have permitted comparisons among populations for decades [7]. A lesson that has emerged is that quality cancer data is possible even in the most limited settings [6]. For registries to flourish and become indispensable to public health, governments must first ensure that surveillance is included as part of their overall health plans so that there is a budget for staff and operations. Unfortunately, too often registries in LMICs are forced to rely on partial, or in some cases complete, *ad hoc* funding, such as from research grants. Without a sustained budget, a registry is vulnerable to staff turnover and a decline in quality.

Closely related to funding is the need for cooperation from clinicians to permit the PBCR to conduct its work. Even when conditions for funding and support are met, it is only sensible to have a registry if there is an accessible health care system. Underlying medical records that document the occurrence of cancer among residents are the primary means of obtaining data for cancer registries. However, the most critical feature for success for cancer registries is a dedicated local leader with technical knowledge in cancer registration. Strong leadership to oversee and nurture the development of the registry is vital to achieve high quality data.

Training, a key component of the GICR, is designed to empower local experts and make use of modern technology. Standardized educational material is developed through local networks of regional trainers to create common sets of teaching slides, references and provide support. The goal is to transfer skills to each trainer, who in turn serves as a resource to further educate registry staff in their respective region. Topics range across all aspects of cancer registry operations, from case finding, coding, management, analyses to reporting. This approach has evolved responsibility for support from a few individuals to a structured, larger group.

There is much still to do; high-quality cancer incidence data is available in less than one-third of countries at present [8]. However, support through the GICR makes the prospects of better cancer data attainable. Based on progress to date, a step-change for at least 30 LMICs in the quality of cancer data is forecasted over the next few years. Linkages between the GICR and the SDGs promote gains that extend beyond cancer to produce a mutual benefit. Stimulating sound practices in surveillance using cancer demonstrates feasibility and can create a greater demand overall for health information.

## Conclusion

Ultimately, the decision on how to proceed resides with each country. The NCD burden will not be tackled without committed and sustainable action by governments. As WHO notes in *World Health Statistics 2019*: “greater investment is needed to improve country health information systems as part of the national statistical system to generate better data, both to inform national decision-making and to reduce reliance on statistical modelling for global monitoring [1]*.*” Plans that are underpinned and monitored by reliable data yields hope of a brighter future. A tried and tested surveillance method for cancer is available to the global oncology community in the form of the PBCR, and ought to be invested in heavily as a means to deliver cancer data for cancer action.

## Conflicts of interest

Neither author reports any conflict of interest.

## Funding statement

Neither author received any funding for this article.
